# Mechanical Performance Enhancement in Natural Fibre-Reinforced Thermoplastic Composites Through Surface Treatment and Matrix Functionalisation

**DOI:** 10.3390/polym17040532

**Published:** 2025-02-18

**Authors:** Ângela Pinto, Dina Esteves, Luís Nobre, João Bessa, Fernando Cunha, Raúl Fangueiro

**Affiliations:** 1 Fibrenamics, Institute of Innovation on Fibre-Based Materials and Composites, University of Minho, 4800-058 Guimarães, Portugal; dinaesteves@fibrenamics.com (D.E.); luisnobre@fibrenamics.com (L.N.); joaobessa@fibrenamics.com (J.B.); fernandocuha@fibrenamics.com (F.C.); rfangueiro@fibrenamics.com (R.F.); 2 Department of Textile Engineering, University of Minho, Azurém Campus, 4800-058 Guimarães, Portugal

**Keywords:** flax fibres, surface treatments, functionalisation, thermoplastic resin, mechanical performance

## Abstract

This study aims to investigate the behaviour of thermoplastic composites reinforced with natural fibres. Composite materials were developed using reactive methyl methacrylate (MMA) resin, commercially known as Elium^®^ (Arkema, Colombes, France), with the incorporation of cellulose nanocrystals (CNCs), dispersed in the matrix at different concentrations. Natural fibres, such as flax, were chemically treated by immersion in an aqueous solution based on NaHCO_3_, during different periods of exposure. After this treatment, flax fibres were washed with distilled water and dried. The degree of fibre surface tension was measured in terms of the contact angle. Then, cellulose nanocrystals were incorporated and mixed in the thermoplastic resin, and the samples were developed via the incorporation of intercalated layers of treated flax fibres. The composites were produced using compression moulding. After that, the samples were evaluated, regarding their mechanical performance and morphology. The research results show that flax fibres treated with 9 wt. % NaHCO_3_ for 48 h had improved flexural strength as a result of removing impurities and exposing hydroxyl groups that react with Na^+^ ions present in NaHCO_3_, which enhances its mechanical properties. The incorporation of 1% CNCs into thermoplastic resin significantly enhanced the fibre/matrix interface, resulting in a remarkable 38% increase in flexural strength. These findings demonstrate the effectiveness of using treated natural fibres and CNCs to improve composites’ performance.

## 1. Introduction

Global concern for sustainability and the environment has propelled the development of products and solutions that minimise environmental impact. As traditional materials raise concerns about resource depletion and pollution, there is a growing demand for eco-friendly alternatives. This shift towards sustainability extends to various industries, including the field of composite materials. Composite materials can generally be defined as heterogeneous materials comprising the synergistic combination of two or more materials [1,2]. The reinforcement and matrix are the two main components of any composite material. The reinforcement serves as the primary load-bearing component, carrying most applied forces. It is characterised by its need for hardness, brittleness, and high strength to effectively bear these loads. Conversely, the matrix acts as a supportive medium, enveloping and safeguarding the reinforcement. The properties of composite materials are influenced by various factors, including the type and characteristics of both the reinforcement and matrix, their arrangement and dispersion within the composite structure, the volume fraction of the reinforcement, and the manufacturing processes employed [3]. The reinforcement constituent is commonly in the form of fibres/fabrics or particles/fillers. As for fibre-reinforced composites (FRCs), they can be reinforced by synthetic fibres or natural fibres. Synthetic fibres have long been used in polymer matrix composites (PMCs) to create lightweight materials that are stronger and more rigid. For example, carbon and glass fibres, two of the most commonly used synthetic fibres, exhibit high mechanical properties, with Young’s modulus values of approximately 230–240 GPa and 70 GPa, respectively. These properties make synthetic fibres highly effective for load-bearing applications [4]. However, the environmental ramifications associated with the production, disposal, and recycling of these synthetic fibre-based composites have prompted a shift towards more sustainable solutions. As a result, there is a growing interest in using natural fibres as an environmentally friendly alternative. Although natural fibres like flax typically exhibit lower mechanical properties (Young’s modulus of about 3.7 GPa), they are increasingly considered for a range of applications across various industries, including automotive and marine components, structural parts, sporting goods, and chemical and construction materials [4].

Natural fibres have many advantageous properties such as their recyclability, eco-friendliness, nominal cost, low density, high specific strength, high impact resistance, high flexibility, and low specific gravity, and they are less abrasive to equipment, have fewer health hazards, are process-friendly, have lower greenhouse emissions, and are CO_2_-neutral [3,5]. Despite all their advantages, there are also some drawbacks associated with the usage of this type of fibre. The hydrophilic nature of natural fibres poses a challenge to their application as reinforcement in polymeric composites [3]. This property leads to poor interfacial adhesion between the fibres and the matrix. The surface of natural fibres comprises oils, cellulose, hemicellulose, lignin, and a waxy layer, all of which pose challenges to achieving effective interfacial bonding. Having strong adhesion between the fibre and matrix plays a crucial role in enhancing both the thermal and mechanical characteristics of the composite [6]. The quality of the interfacial bonding between the fibre and matrix alongside the strength of fibre are parameters that influence the overall strength of composite materials [3].

In composite materials, optimal interfacial bonding between the fibre and the matrix is crucial for maximising the load-bearing capacity of the composite. In practical applications, the loads applied to composite structures are rarely aligned solely with the direction of the fibres; there is almost always a load component at an angle to the fibre axis. In such cases, strong fibre/matrix adhesion is essential to ensure that the maximum level of stress can be maintained across the interface and transferred efficiently from the matrix to the fibres without interruption. If the interfacial bonding is not optimal, the ability of the composite to bear loads is reduced, compromising the overall mechanical performance of the material [7]. Hence, it is critical to enhance the adhesion between the fibre and matrix when producing natural fibre-reinforced composites. Besides the hydrophilic character of natural fibres, other challenges commonly encountered include thermal degradation and biodegradation [8]. To tackle these issues effectively, a range of chemical or physical treatments can be performed to modify the surface of the reinforcing fibre [9]. Some of the chemical treatments known to modify the surface of natural fibres are presented in Table 1.

However, many of the most commonly used reagents for these types of treatments tend to raise health and safety concerns as well as challenges associated with the disposal of hazardous solvents [10,11].

Alternatively, studies report the use of sodium bicarbonate (NaHCO_3_) for natural fibre treatment as a means of achieving better fibre/matrix adherence [12,13,14]. NaHCO_3_ is an environmentally friendly product with a slightly alkaline nature [12,13]. It is commonly used for a variety of purposes, including cooking, gardening, cleaning, and even medicine, without harming public health or the environment [12]. Alkaline treatment, involving the exposure of fibres to an alkaline solution, is a widely used technique for enhancing the properties of natural fibres in composites. This process alters the surface structure of fibres, eliminating non-cellulosic components such as lignin and hemicellulose and revealing cellulose microfibrils, which serve as the primary component responsible for load bearing. This treatment enhances the surface area of the fibre and increases the number of hydroxyl groups, which improve chemical interactions with the matrix [15].

In addition, Das P. et al. conducted a study in which the environmental impact of two surface modification treatments of natural plant fibres was comparatively analysed, one of them, mercerisation, carried out in a sodium hydroxide (NaOH) aqueous solution and the other carried out in a NaHCO_3_ aqueous solution. The results show that the pH of the residual chemical solution following the sodium bicarbonate treatment of the fibres was found to be neutral, suggesting that its disposal poses minimal or no harm to the environment. In contrast, the pH of the sodium hydroxide solution was strongly alkaline, indicating potential adverse effects in disposal areas [13]. Surface modification treatments utilising environmentally friendly products like NaHCO_3_ on natural fibres could hold significant promise for polymeric composites across various applications.

Moreover, in the last few years, fibre-reinforced polymer composites (FRPCs), made by reinforcing polymeric resins with high-strength fibres, have exhibited enhanced and unique properties that are not achievable by either component alone. FRPCs can be manufactured using either thermoset (TS) or thermoplastic (TP) polymeric resins. Recent progress in this area has been achieved with the development of new reactive TP resins such as Elium^®^, Arkema’s novel liquid methyl methacrylate (MMA) thermoplastic resin which shows promising characteristics for various applications. This resin can be cured at room temperature using a mixture of MMA monomer and initiator, presents low-viscosity liquids (100 Pa.s), and is suitable for processing by using liquid composite infusion techniques or compression moulding processes [16]. Furthermore, its low viscosity can facilitate better fibre/matrix bonding, which is crucial for optimising the mechanical properties of composites. Moreover, unlike thermosetting resins, MMA resin can be melted and reprocessed, making it recyclable and environmentally friendly—some of its key features. It is also worth mentioning that MMA resin stands out from other thermoplastic polymers because, unlike them, it can be applied using traditional composite manufacturing procedures without requiring high temperatures. For example, polyamide 6 requires temperatures of about 260 °C for processing, which could degrade natural fibres and fillers, compromising the overall composite’s quality [17]. According to recent research, Elium^®^ is being actively investigated for its recycling ability and can be recycled by two methods, mechanically or chemically, to produce recycled composite parts. In industry, this thermoplastic resin has been utilised in the fabrication of composite sandwich structures, showcasing improved mechanical properties compared to traditional epoxy resins, namely, with significant enhancements in flexural and flatwise strength [18].

Various studies report the use of cellulose particles as nanofillers to reinforce different kinds of composite materials. When the proper dispersion of these fillers within the polymeric matrix is achieved, the mechanical properties of composite materials can be significantly improved. This occurs through uniform stress distribution and the enhanced ability of the fillers to bear and transfer the loads applied to the composite. This approach reduces the stress on the polymeric matrix and consequently enhances the composite’s overall mechanical performance. Additionally, rigid fillers can increase the stiffness of the composite by limiting the mobility and deformation of the polymer chains. As a result, while the composite becomes less flexible, it gains greater resistance to deformation. However, it is crucial to ensure the even dispersion of the fillers; otherwise, points of aggregation can occur which may create weak areas that are prone to failure, ultimately reducing the performance of the composite [16,18,19,20,21]. In the field of composite production, nanocellulose has emerged as a prominent choice for reinforcing polymer matrices. These composites consist of polymeric matrices fortified with nanocellulose fillers. The utilisation of nanocellulose crystals in this role has attracted considerable interest owing to their intrinsic properties and potential applications. Cellulose is a material extracted from abundantly available natural resources such as plants, algae, fungi, and bacteria. It is characterised by being the most affluent bio-based polymer, eco-friendly, toxic-free, and biodegradable [22]. Such properties make it compelling to be explored extensively in the preparation of biomaterials, namely bio-composites.

The purpose of the present paper is to study the hypothesis that sustainable thermoplastic composites can be developed by reinforcing MMA-based thermoplastic resins with CNCs and natural fibres, specifically flax, treated through an environmentally friendly chemical process using NaHCO₃. This treatment is expected to enhance fibre/matrix adhesion without significant environmental impact. Additionally, this study postulates that the incorporation of CNCs at varying concentrations will further improve the mechanical performance of composites. By optimising the duration of the fibre treatment and the CNC concentration, this research aims to achieve a balance between sustainability and enhanced composite properties.

## 2. Materials and Methods

### 2.1. Raw Materials

The natural fibres selected for this experimental study consisted of a non-crimp unidirectional flax fabric with fibres oriented at 0°, featuring 150 g/m^2^ of mass per unit area, supplied by Bcomp, Switzerland. The flax fibres were treated using NaHCO_3_ purchased from Nogueira—Materiais de Construção, Guimarães, Portugal. Some physical and mechanical properties of the flax fibres are summarised in Table 2.

The acrylic polymer used for the composite preparation was a liquid reactive MMA thermoplastic resin (Elium^®^ 151 XO/SA), supplied by Arkema, Colombes, France. The Elium^®^ 151 XO/SA resin system is made of 3 components with 151 XO resin, a 151 SA accelerator, and a peroxide initiator. When all 3 components are mixed, radical polymerisation occurs to produce a poly(methylmethacrylate) (PMMA) TP matrix. The initiator used in this research work was Butanox^®^ M-50, a medium-reactive, general-purpose methyl ethyl ketone peroxide (MEKP), a solution in dimethyl phthalate, purchased from Ecocompósitos, SA, Faro Portugal. The typical cured non-reinforced Elium^®^ resin properties are detailed in Table 3.

The cellulose nanocrystals (CNCs) used to functionalise the TP matrix were supplied by CelluForce, Montreal, QC, Canada. These nanoscale (10^−9^ m) particles are derived from cellulose, a natural polymer found in plant cell walls, and have a bulk density of 1300 g/cm^3^ and a moisture content of approximately 4%. When dispersed at 2% *w*/*w* in deionised water, their particle size is typically around 0.2 µm, with a conductivity of 225 µS/cm and a pH of 7.

### 2.2. Surface Treatment of Flax Fibres

NaHCO_3_ is capable of dissolving in water at concentrations of up to approximately 10% [13]. Previous studies have reported the use of NaHCO_3_ concentrations ranging from around 3% to 10% for treating natural fibres in order to modify their surface properties [13,15,23]. In particular, a study conducted by Das P. et al. demonstrated significant improvements in the mechanical properties of the composite after treating the reinforcing natural fibres using a concentration of 9% NaHCO_3_ [13].

The duration of the treatment with sodium bicarbonate (NaHCO_3_) of the flax fibres was selected on the basis of previous studies and with the aim of investigating the influence of the treatment time on the properties of the fibres. Studies have reported treatment times ranging from 4 h to 120 h with better results observed for longer treatment times [13,15,23]. To assess the effects of exposure time to NaHCO_3_ on the chemical and physical changes to the fibres, as well as on the final properties of the composites produced, the selected treatment times were 24, 48, 72, and 96 h.

For this test, the flax fabric was dipped in an aqueous solution of NaHCO_3_ at a concentration of 9 wt. % for the different periods of time selected (24, 48, 72, and 96 h). The treatment was carried out at room temperature. Once the treatment times elapsed, the fibres were removed from the treatment solution and rinsed in distilled water. The samples were then dried in an oven at 50 °C for approximately 18 h.

**Figure 1:**
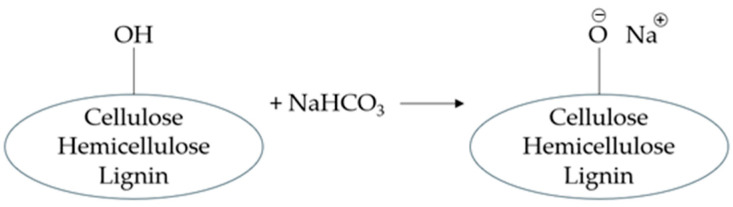
Reaction between flax fibres and sodium bicarbonate [24].

Figure 1 illustrates the chemical reaction occurring during the treatment of flax fibres with sodium bicarbonate, which contributes to the enhancement in mechanical properties.

The dissolution of NaHCO_3_ in aqueous solution results in the formation of Na⁺ and HCO3- ions. These ions interact with the hydroxyl groups (OH) present in the fibres, as illustrated in the chemical reaction shown in Figure 1. The Na⁺ ion replaces the proton of the hydroxyl group, forming an ionic bond between oxygen in the fibre and sodium.

This interaction leads to the replacement of hydrophilic hydroxyl groups, producing fibres with modified surface properties [25].

The effectiveness of the treatment was assessed through the analyses of the fibres’ contact angles as well as with flexural testing performed on the resulting composites, which will be discussed in detail in the subsequent sections.

### 2.3. Matrix Functionalisation and Composite Sample Preparation

To functionalise the matrix, CNC powder was first dried in an oven at 60 °C for 24 h. Following this, the CNCs were mixed into the MMA resin at varying weight percentages (1.0%, 2.5%, and 5.0%) and stirred manually for 5 to 10 min until no lumps are visible. The resulting suspension underwent ultrasonication using an Ultrasonic Cleaners serie EP S3, from Soltec, Milan, Italy, at 40 kHz frequency and 180 W power for 30 min. A calculated amount of 1.5 wt. % of initiator was then added to the CNC-dispersed resin suspension, taking into account the used resin quantity. In the preparation of composites, 12 layers of flax fabric orientated according to the directional angles [−45°/0°/45°/−45°/90°/45°/−45°/0°/45°/−45°/90°/45°], corresponding to 40% of the total weight of the final composite, were impregnated with the Elium^®^ 151 SA/XO resin or the CNC-dispersed resin suspension using a hand lay-up technique. This layered structure was then placed in a compression moulding press (LabManual 300, Fontijne, Rotterdam, The Netherlands) at 60 °C for 10 min at 1 bar. Subsequently, the pressure was increased to 9 bars for 35 min to prepare the final samples, as described in Table 4.

### 2.4. Contact Angle

To verify whether the treatment influenced the hydrophilicity of the flax fibres, the fibres were subjected to contact angle measurements according to the ASTM D7334-08 standard [26]. Treated and untreated flax fibres had their contact angles estimated at room temperature using an optical contact angle goniometer. This involved using a Data Physics Contact Angle OCA 200 goniometer (DataPhysics Instruments GmbH, Filderstadt, Germany) to place 10 drops of water of 5 µL volume each, at a flow rate of 10 µL/s, at random spots on each flax fabric sample. The average contact angle for each sample was calculated from the multiple measurements at room temperature to ensure the accuracy and reliability of the results. The contact angles were then recorded and estimated immediately after drop contact with the surface, using the goniometer video drop shape analyser OCA 15 plus software (version 1.2). Special care was taken to ensure that the fibres were laid flat and free of wrinkles or folds that could affect the accuracy of the measurements.

### 2.5. Thermogravimetric Analysis (TGA)

TGA was conducted on the untreated and treated flax fibre samples in order to evaluate the influence of NaHCO_3_ surface treatment on the thermal stability of flax fibres using an Hitachi STA 7200 (Hitachi, Tokyo, Japan). Untreated and treated fibres underwent a heating process from room temperature to 600 °C with a heating rate of 10 °C/min under nitrogen flow.

### 2.6. Fourier-Transform Infrared (FTIR) Spectroscopy

The chemical composition of the flax fibre samples was assessed by ATR-FTIR analysis utilising IRAffinity-1S SHIMADZU equipment (Kyoto, Japan). Spectra were acquired with a diamond ATR crystal cell by the accumulation of 45 scans with a resolution of 8 cm^−1^ from 400 to 4000 cm^−1^.

### 2.7. Flexural Testing

Flexural testing was carried out according to the EN ISO 178:2003 standard [27] (equivalent to ASTM D790-17 [28]), using an H100KS Hounsfield Universal Testing Instrument (Hounsfield, RedHill, UK). The test was conducted at room temperature with a 5 kN load cell and a crosshead speed of 5 mm/min. Five specimens of each sample were tested. Various properties of the flexural stress/strain relationship were determined, including flexural strength, flexural modulus, and flexural strain.

### 2.8. Scanning Electron Microscopy (SEM) Analysis

Lastly, for morphological and microstructural analyses, all composite samples were examined using a Leica M125 microscope (Leica Microsistemas Lda., Carnaxide, Portugal) and a Hitachi FlexSEM 1000 II scanning electron microscope (SEM) (Hitachi, Tokyo, Japan). The samples were prepared for analysis by applying a thin coating (20 nm) of a Gold (Au) and Palladium (Pd) alloy, using a Leica EM ACE200 (Leica Microsistemas Lda., Carnaxide, Portugal) vacuum coater, and were examined under an acceleration voltage of 10 kV.

## 3. Results and Discussion

### 3.1. Contact Angle

**Figure 2:**
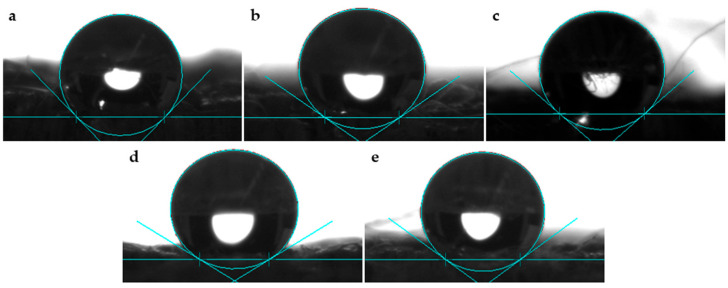
Optical photographs of water droplets on surface of flax fabric samples after treatment with NaHCO₃, showing contact angles: (**a**) untreated control flax fabric sample (F), (**b**) flax fabric sample treated with NaHCO_3_ for 24 h (FA924), (**c**) flax fabric sample treated with NaHCO_3_ for 48 h, (**d**) flax fabric sample treated with NaHCO_3_ for 72 h (FA972), and (**e**) flax fabric sample treated with NaHCO_3_ for 96 h (FA996).

**Figure 3:**
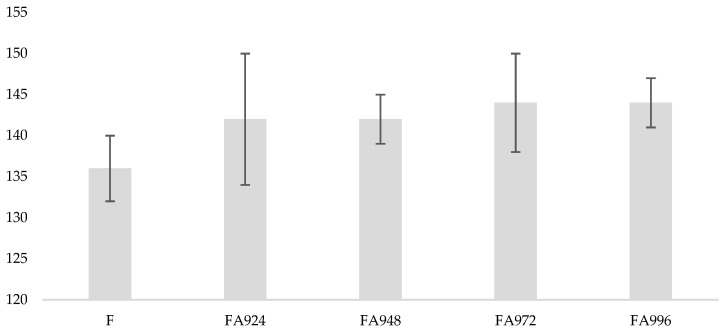
Contact angle (CA) of flax fibres before and after treatment with NaHCO₃, measured after 24 (FA924), 48 (FA948), 72 (FA972), and 96 (FA996) hours.

Figure 2 shows optical photographs of the water droplets on the surface of the flax fabric samples. The quantitative results obtained from measuring the contact angle of flax fibres before and after treatment with NaHCO_3_ are shown in Figure 3.

The results show that the untreated flax fibres exhibited the lowest contact angle value (136°). After treatment with NaHCO_3_, the contact angle progressively increased over time. The 24 and 48 h treated fibres had a contact angle of 142°, and this value continued to increase slightly, reaching 144° in the samples treated for 72 and 96 h.

These results indicate a slight increase in the hydrophobicity of flax fibres after treatment with NaHCO_3_, whereby the longer the treatment time, the greater the contact angle. The preparation of an aqueous NaHCO_3_ solution involves the formation of carbonic acid (H_2_CO_3_) and a hydroxide ion (OH^−^), which makes it slightly alkaline. Additionally, based on the predominance of alcoholic hydroxyl groups (which are weak acids) in the fibres, it is reasonable to suggest that the interaction resembles what occurs in a conventional mercerisation treatment [29]. For this reason, it is plausible to assume that similarly to mercerisation, the NaHCO_3_ pre-treatment reduces the fibre diameter, which increases the aspect ratio, which leads to the development of a rough surface topography [29,30]. The increase in the roughness of the fibre surface may explain the increase in the contact angle.

On the other hand, this test showed that although the flax fibres treated with NaHCO_3_ had a higher contact angle than the untreated flax, the water droplets were absorbed over time, but for the untreated flax, despite having a slightly lower contact angle, the droplets remained on the sample without being absorbed, regardless of the time elapsed. Fourier-transform infrared spectroscopy (FTIR) analyses carried out by Fiore V. et al. on sisal fibres before and after treatment with an aqueous NaHCO_3_ solution revealed that by pre-treating lignocellulosic fibres with NaHCO_3_, it was possible to achieve the removal of hemicellulose and a reduction in lignin from the surface of the fibre [29]. The removal of these compounds, as well as impurities existent on the fibres, usually results in an improvement in the fibres’ ability to absorb moisture, which may explain the phenomenon described above.

### 3.2. FTIR

**Figure 4:**
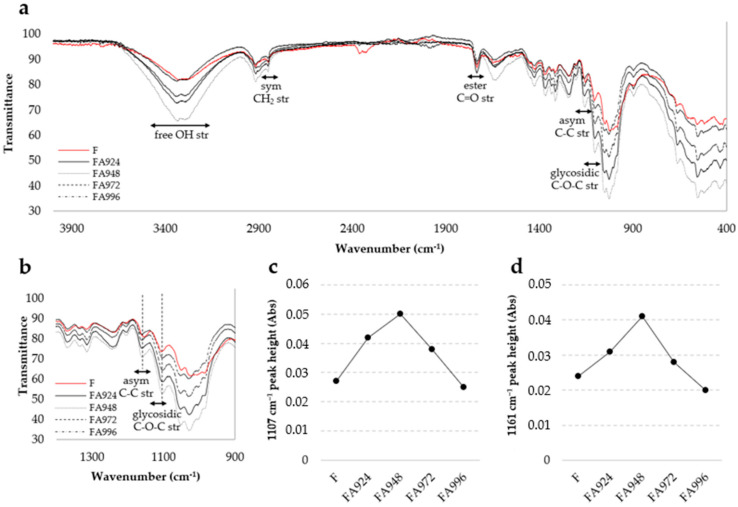
FTIR spectra of flax samples. (**a**) FTIR spectra of untreated (F) and treated fibres for 24 (FA9249), 48 (FA948), 72 (FA972), and 96 (FA996) h; (**b**) selected region of spectra for clearer visualisation of cellulose peaks of interest; (**c**) peak height of FTIR spectra at 1107 cm^−1^ as function of duration of treatment; and (**d**) peak height of FTIR spectra at 1161 cm^−1^ as function of duration of treatment.

The FTIR spectra of untreated and NaHCO_3_-treated flax fibres are shown in Figure 4.

The FTIR analysis of both treated and untreated flax fibres revealed bands typical of this material [31,32,33,34]. A band was identified around 3335 cm^−1^, related to the stretching of free OH groups. Another band, at 2850 cm^−1^, corresponded to the symmetrical stretching of CH_2_ groups, common in the methylene groups of organic compounds. The band around 1735 cm^−1^ was associated with the stretching of the C=O bond, characteristic of the ester groups present in pectins. Near 1505 cm^−1^, there was a band attributed to the stretching of the C=C bond in the aromatic rings of lignin. A band was also observed around 1650 cm^−1^, linked to the water adsorbed by the fibres. Finally, other relevant bands appeared close to 1155 cm^−1^ and 1105 cm^−1^, attributed to the stretching of the C–C bond and the C-O-C glycosidic bond, both characteristic of cellulose.

Furthermore, for the CF sample, a band was observed at around 2300–2400 cm^−1^, likely resulting from artefact peaks from gaseous CO_2_ [35]. It is assumed that the disappearance of this band in the treated samples is probably due to variations in the amount of CO_2_ present in the optical path during the FTIR measurements.

Additionally, in the spectra of sample FA972, a subtle peak was detected around 1950 cm⁻^1^. This region does not correspond to any known functional groups commonly reported in flax fibres. For this reason, we believe that this peak is not indicative of a new functional group but rather an artefact arising from the measurement process or potential environmental interference during the acquisition of the spectra.

The spectra obtained show more intense bands for the fibres treated for 48 h (FA948), namely the bands attributed to cellulose (Figure 4b–d), suggesting the existence of more chemical groups associated with these bands. Conversely, the untreated flax fibre sample (F) and the 96 h NaHCO_3_-treated sample (FA996) show spectra with the least intense bands, suggesting that there are fewer of the same chemical groups.

### 3.3. TGA

**Figure 5:**
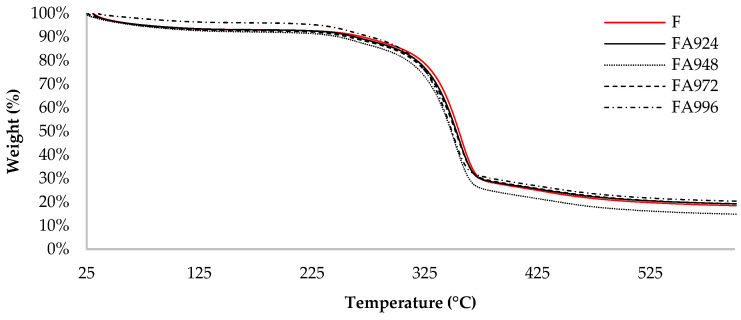
TGA curves of untreated (F) and NaHCO_3_-treated flax fibre samples for 24 (FA924), 48 (FA948), 72 (FA972), and 96 (FA996) h.

**Figure 6:**
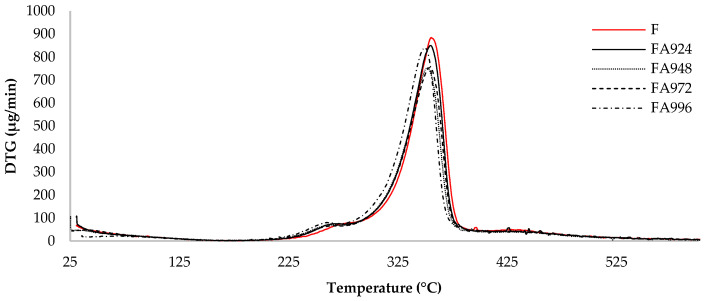
DTG thermograms of untreated (F) and NaHCO_3_-treated flax fibre samples for 24 (FA924), 48 (FA948), 72 (FA972), and 96 (FA996) h.

The results of the thermogravimetric analysis of the flax fibres are shown in Figure 5 and Figure 6.

TGA revealed results characteristic of flax fibres, with mass loss profiles and degradation temperatures typical of this material [31,32,34,36].

The TGA curves show that the onset temperature of degradation for all the samples was close to 200 °C, with the majority of degradation occurring up to 385 °C. It was also observed that the sample FA948 had the lowest percentage of residue at 600 °C (15%), indicating a lower amount of compounds resistant to this temperature in its composition. On the other hand, the sample FA996 had the highest amount of residue (20%) at the end of the test. The remaining samples showed similar results, with samples FA924 and FA972 showing 19% residue, while the control sample (F) showed 18% residue.

In the DTG thermogram, a small initial peak, observed in the 30 °C to 150 °C range, is related to the loss of mass due to the evaporation of the water present in the fibres. A small shoulder can then be seen between 200 °C and 270 °C, which can be attributed to the decomposition of hemicellulose and pectin. In addition, for all the samples, a main peak is observed around 350 °C, which corresponds to the degradation of cellulose.

Although no significant differences were observed between the treated and untreated samples, the peaks of samples FA948 and FA972 show lower DTG values (μg/min) compared to the others, especially the control sample (F). This means that the material is losing mass more slowly in that temperature range, suggesting that the material is more resistant to thermal decomposition at that temperature.

The results obtained show that the surface treatment applied to the fibres did not have a significant impact on their thermal resistance properties, as evidenced by the TGA and DTG curves. However, small differences can be observed at specific temperatures, such as a slight reduction in the thermal resistance of the fibres after treatment. This observation aligns with the behaviour reported by Raharjo et al., where untreated fibres exhibited marginally higher thermal stability compared to treated ones, indicating that the NaHCO_3_ treatment can slightly reduce the processing temperature [25]. This effect is attributed to the removal of non-cellulosic components, such as lignin and hemicellulose, during the treatment process. While the elimination of these components enhances fibre/matrix adhesion, it can also lead to a minor decrease in the overall thermal stability of the fibres.

### 3.4. Flexural Properties

Flexural testing was conducted on each thermoplastic composite reinforced with untreated flax fibres and flax fibres subjected to different NaHCO_3_ treatment times (24, 48, 72, and 96 h). Table 5 shows the flexural strength, flexural modulus, and flexural deformation values obtained for each sample.

The results show a considerable variation in mechanical properties between the samples. The flexural strength ranged from 72.1 MPa (CF), corresponding to the untreated material, to 157.5 MPa, with sample CFA948_1CNC showing the highest value. Samples CFA948, CFA948_2.5CNC, and CFA948_5CNC also exhibited high strength, with values of 114.1 MPa, 125.9 MPa, and 115.3 MPa, respectively.

Regarding the flexural modulus, the values ranged from 2.8 MPa (CF) to 15.8 MPa, again with sample CFA948_1CNC standing out with the highest value. Samples CFA948 and CFA948_5CNC also showed higher flexural modulus values, exceeding 4 MPa, indicating greater rigidity compared to the other samples.

Flexural deformation ranged from 2.1% (CFA948_1CNC) to 7.8% (CFA996). Samples CF and CFA996, which both showed lower flexural strength and flexural modulus, exhibited the highest deformations, at 7.5% and 7.8%, respectively. Conversely, samples CFA948 and CFA948_1CNC showed deformations lower than 5%, suggesting a more rigid and less deformable behaviour.

The composite sample reinforced with the fibres treated for 48 h (CFA948) showed a significant 58% improvement in flexural strength compared to the composite sample produced with untreated fibres (CF), when the flax fibres were treated with 9 wt. % NaHCO₃. However, a noticeable decrease in flexural strength and flexural modulus, along with an increase in flexural strain, was observed in the composite samples developed with the flax fibres treated for 72 and 96 h (samples CFA972 and CFA996, respectively). Therefore, it can be inferred that prolonged NaHCO*3* exposure can result in the degradation of the fibre, leading to a reduction in cellulose content [24]. Since cellulose is a key component of flax fibre, being responsible for its ability to withstand mechanical loads, its loss weakens the overall fibre structure. This can compromise the mechanical integrity of the reinforcing fibre and, therefore, its ability to transfer loads efficiently within the matrix. Moreover, the damage of the fibre surface can impair effective bonding with the matrix, further compromising adhesion. These alterations can collectively result in a decrease in the mechanical properties of the produced composites.

Since sample CFA948 achieved the best mechanical performance, its properties were further studied by functionalising the matrix with CNC incorporation. The CFA948_1CNC, CFA948_2.5CNC, and CFA948_5CNC samples, which underwent matrix functionalisation, exhibited a significant improvement in mechanical properties. This improvement was particularly notable for sample CFA948_1CNC, functionalised with 1.0 wt. % CNC, which demonstrated a 38% increase in flexural strength, a 172% increase in the flexural modulus, and a 119% reduction in flexural strain compared to sample CFA948, without matrix functionalisation.

The mechanical performance of the samples improved significantly with the addition of CNCs, primarily due to enhanced interactions within the matrix and improved fibre/matrix interfacial interactions. The dispersion of CNCs within the Elium^®^ resin significantly enhanced the compatibility between flax fibres and the matrix, resulting in improved fibre/matrix interfacial properties.

However, for CNC concentrations equal to or greater than 2.5 wt. % (samples CFA948_2.5CNC and CFA948_5CNC), a decline in mechanical performance was observed compared to CFA948_1CNC. This reduction in performance is attributed to potential CNC agglomeration at higher concentrations, leading to non-uniform dispersion within the polymer matrix. Such agglomeration can result in localised stress concentrations and weak points, ultimately compromising the mechanical integrity of the composites [19,22].

### 3.5. Scanning Electron Microscopy (SEM) Analysis

An analysis of the scanning electron microscopy (SEM) images shown in Table 6 reveals significant differences in surface quality and adhesion between the flax fibres and the matrix as a function of NaHCO₃ treatment and exposure duration.

In general, it was found that adhesion between fibres and the matrix improved after treating the fibres with NaHCO_3_. Sample CF, which received no treatment, had worse surface quality and poor adhesion between the fibres and the matrix, as shown in Figure 4a,b. Gaps and fractures can be seen between the fibres and the matrix, indicating low interfacial interaction, which suggests a greater presence of impurities on the surface.

After treatment with NaHCO_3_, samples exhibited improved surface quality, indicating better adhesion between the fibres and the matrix. This effect is especially noticeable in sample CFA948, which had fibres exposed to the treatment for 48 h, where the interface between the fibres and the matrix shows a more homogeneous bond and fewer visible defects compared to sample CF. However, an increase in the exposure time to NaHCO_3_ to 96 h appears to result in a decrease in the adhesion between the flax fibres and the matrix, especially compared to sample CFA948. This decrease in adhesion suggests that prolonged treatment times can have an adverse effect on the interface.

In the SEM images, a noticeable difference can be observed when comparing the control sample (CF) with the samples produced using NaHCO_3_-treated fibres, particularly in the cross-sectional views. After the treatment, a greater amount of resin adhering to the fibres is evident, along with a reduced presence of fibres not fully covered by resin. This suggests an improved fibre/matrix interaction and better resin penetration as a result of the NaHCO_3_ treatment. The improved fibre/matrix interaction can be attributed to the increased surface roughness and potential chemical modifications on the fibre surface caused by the NaHCO_3_ treatment.

Analysing the images of the samples whose matrix was functionalised (CFA948_1CNC, CFA948_2.5CNC, and CFA948_5CNC) revealed the presence of particles that are likely to be CNCs. Specifically, the cross-sectional images of sample CFA948_5CNC show a marked stiffness in the fibres. The presence of a greater quantity of cellulose nanocrystals (CNCs) dispersed in the matrix is visible in this sample, which gives it a more rigid and less malleable structure.

## 4. Conclusions

This study demonstrates the potential for improving the mechanical properties of thermoplastic composites reinforced with natural fibres, specifically using flax fibres treated with NaHCO₃ and the incorporation of cellulose nanocrystals (CNCs) into the thermoplastic resin matrix. Treating the flax fibres with NaHCO₃ resulted in a significant improvement in fibre/matrix adhesion, especially with the 48 h treatment. This treatment contributed to a significant 58.25% increase in flexural strength compared to the untreated flax fibre material.

Furthermore, the addition of CNCs to the thermoplastic resin matrix provided a significant increase in the mechanical properties of the composites, with the sample functionalised with 1.0 wt. % of CNCs standing out, with a 38% increase in flexural strength, a 172% increase in the flexural modulus, and a 119% reduction in flexural deformation. The results indicate that functionalising the matrix with CNCs results in composites with better mechanical properties. However, higher concentrations of CNCs (2.5% and 5.0%) led to the formation of agglomerates, which compromised the mechanical integrity of the material.

Morphological analyses by SEM confirmed improvements in adhesion between the fibres and the matrix, especially in samples treated with NaHCO₃ for 48 h. However, a decrease in adhesion was observed with prolonged treatments of more than 72 h, indicating that excessive treatment times can be counterproductive.

These results indicate that both the chemical treatment of natural fibres with NaHCO_3_ and the functionalisation of the matrix with CNCs are effective and sustainable strategies for improving the mechanical performance of natural fibre-reinforced composites. These advances are relevant in the context of the high demand for sustainable solutions and in line with efforts to reduce the environmental impact of industrial materials.

## Figures and Tables

**Table 1:** Commonly used treatments for the modification of the surface of natural fibres (adapted from Jothi Arunachalam et al., 2024) [10].

Treatment	Result
Mercerisation (Sodium Hydroxide Treatment)	Removal of hemicellulose, lignin, pectin, and waxy layers, causing exposure of cleaner fibre surface.
Acetic Acid Treatment	Enhancement in mechanical characteristics and thermal stability of fibres.
Silane Treatment	Increase in both mechanical and thermal performance of natural fibres.
Benzoyl Peroxide Treatment	Facilitates interaction between hydrophilic fibres and hydrophobic matrices.
Phosphoric Acid Treatment	Alteration in fibre’s physical structure by removing pectin, oil, and waxy components.
Sulphuric Acid Treatment	Efficient improvement in both physical and chemical properties of fibres.
Peroxide Treatment	Enhancement in compatibility between natural fibres and polymer matrices.
Isocyanate Treatment	Substantial improvement in fibre strength and decrease in water absorption.

**Table 2:** Physical and mechanical properties of flax fibres.

Properties	Value
Fibre density	1.47 ± 0.02
Apparent modulus, GPa	62 ± 2
Elongation at break	1.3–1.4%
Water content *	5–6%

* Valid at ambient conditions: 22 °C, 50% RH.

**Table 3:** Typical cured non-reinforced Elium^®^ 151 XO/SA resin properties.

Properties	Value
Heat Deflection Temperature, °C	75.90
Tensile Strength, MPa	47.10
Tensile Modulus, GPa	2.68
Tensile Elongation@break	4.50%
Flexural Strength, MPa	80.51
Flexural Modulus, GPa	2.77
Flexural Elongation@MaxStrength	4.42

**Table 4:** Plan of experiments.

Fibre Sample Code	Fibre Surface Treatment	Composite Sample Code	Matrix Functionalisation
F	—	CF	—
FA924	9 wt. % NaHCO_3_ for 24 h	CFA924	—
FA948	9 wt. % NaHCO_3_ for 48 h	CFA948	—
FA972	9 wt. % NaHCO_3_ for 72 h	CFA972	—
FA996	9 wt. % NaHCO_3_ for 96 h	CFA996	—
FA948	9 wt. % NaHCO_3_ for 48 h	CFA948_1CNC	1.00 wt. % CNCs
FA948	9 wt. % NaHCO_3_ for 48 h	CFA948_2.5CNC	2.50 wt. % CNCs
FA948	9 wt. % NaHCO_3_ for 48 h	CFA948_5CNC	5.00 wt. % CNCs

**Table 5:** Mechanical performance of polymer composites reinforced with flax fibres, showing flexural strength, flexural modulus, and flexural strain for each composite sample.

Sample	Flexural Strength (MPa)	Flexural Modulus (MPa)	Flexural Strain (%)
CF	72 ± 4	2.8 ± 0.1	7.5 ± 0.4
CFA924	92 ± 15	3.6 ± 0.6	5.9 ± 0.9
CFA948	114 ± 13	5.8 ± 0.3	4.6 ± 0.3
CFA972	82 ± 13	4.1 ± 0.5	5.2 ± 0.8
CFA996	77 ± 5	2.9 ± 0.1	7.8 ± 0.9
CFA948_1CNC	157 ± 11	16.00 ± 2	2.1 ± 0.2
CFA948_2.5CNC	126 ± 21	4.0 ± 0.7	5.1 ± 0.6
CFA948_5CNC	115 ± 4	4.3 ± 0.5	5.0 ± 0.7

**Table 6:** SEM images of surface and cross-section of all composite samples.

Sample	Surface	Cross-Section
CF	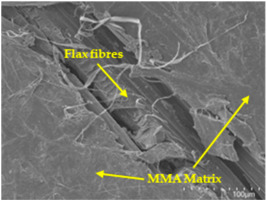	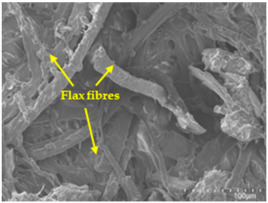
CFA924	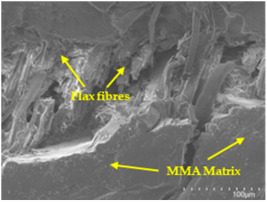	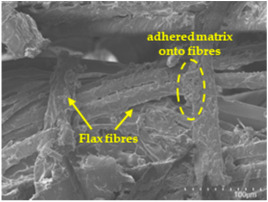
CFA948	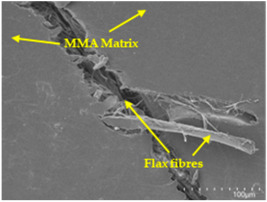	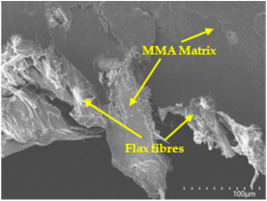
CFA972	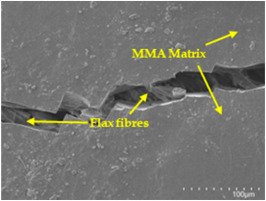	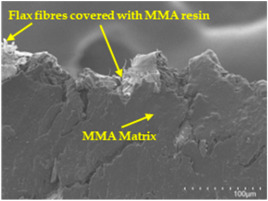
CFA996	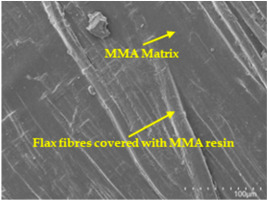	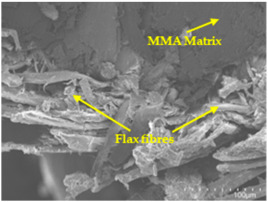
CFA948_1CNC	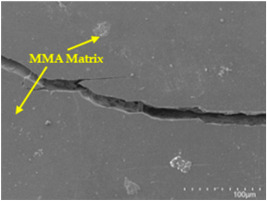	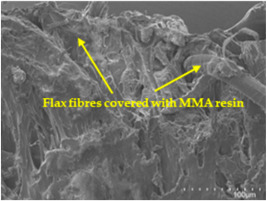
CFA948_2.5CNC	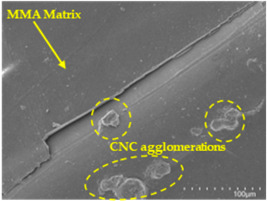	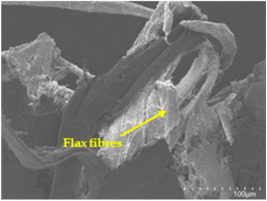
CFA948_5CNC	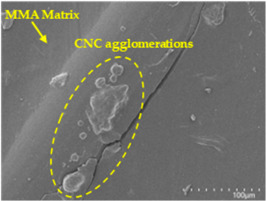	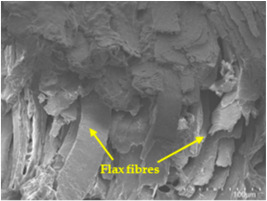

## Data Availability

All data are contained within this article.
